# A cyclometalated iridium(III) complex induces paraptotic cell death via mitochondrial dysfunction and ER stress in triple-negative breast cancer cells

**DOI:** 10.3389/fphar.2026.1739226

**Published:** 2026-01-26

**Authors:** Houmin Lin, Jianhua Wei, Wenmin Yao, Qinqin Zhang, Junfei Jin

**Affiliations:** 1 Guangxi Key Laboratory of Molecular Medicine in Liver Injury and Repair, The First Affiliated Hospital of Guilin Medical University, Guilin, Guangxi, China; 2 Guangxi Health Commission Key Laboratory of Basic Research in Sphingolipid Metabolism Related Diseases, The First Affiliated Hospital of Guilin Medical University, Guilin, Guangxi, China; 3 China-USA Lipids in Health and Disease Research Center, Guilin Medical University, Guilin, Guangxi, China; 4 Department of Thyroid and Breast Surgery, Nanxishan Hospital of Guangxi Zhuang Autonomous Region, Guilin, Guangxi, China

**Keywords:** er stress, iridium(III) complex, mitochondrial dysfunction, paraptosis, TNBC

## Abstract

**Background:**

Given the lack of targeted therapies and frequent resistance to apoptosis-based treatments, triple-negative breast cancer (TNBC) remains a major clinical challenge. Exploring non-apoptotic cell death mechanisms may offer new therapeutic avenues to circumvent drug resistance in TNBC.

**Methods:**

The anticancer activity of a novel cyclometalated iridium (III) compound, CIr2, was evaluated using cytotoxicity, clonogenic, and migration assays in multiple breast cancer cell lines. Mechanistic investigations included analyses of mitochondrial dysfunction, reactive oxygen species (ROS) production, ATP depletion, endoplasmic reticulum (ER) stress, and MAPK signaling. Transcriptomic profiling (RNA-seq), ultrastructural and morphological analyses, as well as pharmacological inhibitor studies targeting distinct cell death pathways, were performed to elucidate the mode of cell death induced by CIr2. The *in vivo* antitumor efficacy and safety of CIr2 were further assessed using a TNBC xenograft mouse model.

**Results:**

CIr2 selectively inhibited the proliferation and migration of TNBC cells while exerting minimal cytotoxic effects on normal breast epithelial cells. CIr2 preferentially accumulated in mitochondria, leading to mitochondrial membrane potential collapse, excessive ROS production, and profound ATP depletion. Transcriptomic profiling and morphological analyses revealed pronounced ER stress, MAPK pathway activation, and paraptosis-associated ultrastructural alterations, including mitochondrial swelling and extensive cytoplasmic vacuolization. Pharmacological inhibition of apoptosis, necroptosis, ferroptosis, autophagy, ER stress, or p38 MAPK signaling failed to rescue CIr2-induced cytotoxicity, whereas ROS scavenging effectively reversed these effects, confirming a mitochondrial dysfunction and ROS-driven paraptotic mode of cell death. *In vivo*, CIr2 markedly suppressed TNBC xenograft tumor growth with minimal systemic toxicity.

**Conclusion:**

CIr2 induces paraptosis through mitochondrial dysfunction and ER stress, offering a potential therapeutic strategy to overcome apoptosis resistance in TNBC. These findings provide a new mechanistic insight into iridium-based paraptosis induction.

## 1 Introduction

Breast cancer is a heterogeneous disease characterized by several molecular subtypes. Of these, TNBC is notable for its absence of estrogen receptor (ER), progesterone receptor (PR), and human epidermal growth factor (HER2) expression (Obidiro et al., 2023). The lack of molecular targets renders TNBC largely unresponsive to current targeted therapies, leading to poor clinical outcomes and a high risk of metastasis (Maqbool et al., 2023). A major challenge in TNBC therapy is its frequent resistance to apoptosis, the main mechanism by which conventional chemotherapeutic agents eliminate cancer cells (Ferrari et al., 2022; Goodwin et al., 2015; Nedeljković and Damjanović, 2019; Azari et al., 2023). This highlights the urgent need for alternative therapeutic strategies that can activate non-apoptotic cell death to circumvent therapeutic resistance (Malla et al., 2024; Sepand et al., 2020).

Paraptosis is a non-apoptotic form of programmed cell death. It has recently emerged as a promising alternative mechanism for targeting apoptosis-resistant cancers. In contrast to the nuclear fragmentation and apoptotic body formation typical of apoptosis, this process features extensive cytoplasmic vacuolation and swelling of the mitochondria and ER. Paraptosis is closely linked to mitochondrial dysfunction, ER stress, and MAPK signaling activation (Xu et al., 2024; Che et al., 2024; Hanson et al., 2023; Fontana et al., 2020). Nevertheless, the molecular regulation of paraptosis remains poorly understood, and only a limited number of small-molecule inducers have been discovered. Therefore, investigating novel pharmacological compounds capable of inducing paraptosis may yield important insights into the development of next-generation anticancer agents. However, whether cyclometalated iridium (III) complexes can induce paraptosis in TNBC remains unknown.

Metal-based complexes exemplified by cisplatin have been pivotal in cancer therapy (Zhang et al., 2022; Alassadi et al., 2022). Nevertheless, drug resistance and severe systemic side effects often limit their clinical application (Rottenberg et al., 2021; Lv et al., 2021; Shahlaei et al., 2023), thereby promoting the development of new metal complexes that operate through alternative mechanisms. Iridium (III) complexes have recently gained attention due to their remarkable stability, versatile photophysical, redox properties, and ability to interact with diverse intracellular targets (Lo and Zhang, 2012; Guan et al., 2020; Ho et al., 2020; Szymaszek et al., 2024). Several iridium-based compounds have been reported to induce apoptosis, autophagy, ferroptosis, and pyroptosis in various cancer models (He et al., 2023; Chen et al., 2021; Li et al., 2025; Lu et al., 2025; Hu et al., 2024), yet their capacity to trigger paraptosis remains largely unexplored.

In our earlier work, we synthesized CIr2, a cyclometalated Ir(III) complex that exhibited strong anticancer effects in pancreatic cancer cells through triggering apoptosis (Lin et al., 2024). Building on these results, we further investigated the impact of CIr2 in breast cancer cells and observed that it selectively inhibits the proliferation and migration of TNBC cells. Mechanistically, CIr2 was demonstrated to induce caspase-independent cell death, characterized by mitochondrial dysfunction, ER stress, and p38 MAPK activation, consistent with the hallmarks of paraptosis. Moreover, in a TNBC xenograft model, CIr2 dramatically inhibited tumor growth with minimal systemic toxicity. Although several chemical agents have been reported to induce paraptosis (Seo et al., 2023; Dai et al., 2021; Xue et al., 2018; Hu et al., 2023), this is the first study showing that a cyclometalated Ir(III) complex can induce paraptosis in TNBC cells. These findings reveal a novel pharmacological mechanism of iridium-based anticancer compounds and provide a new therapeutic avenue for overcoming apoptosis resistance in TNBC.

## 2 Materials and methods

### 2.1 Materials and reagents

CIr2 was synthesized by our group as previously described (Lin et al., 2024). The following reagents were procured from commercial suppliers: CCK-8, N-acetyl-L-cysteine (NAC), staurosporine, Z-VAD-FMK, necrostatin-1, SB202190, cisplatin, ATP Assay Kit (#S0026), Mito-Tracker Red (#C1049B), ROS Assay Kit (#S0033S), Apoptosis Detection Kit (#C1062S), tetramethylrhodamine ethyl ester (TMRE) were obtained from Beyotime Biotechnology (China). Ferrostatin-1 was acquired from MedChemExpress (USA). CB-5083 was purchased from TargetMol (USA). Sodium 4-Phenylbutyrate (4-PBA), Hydroxyurea (HU), Hydroxychloroquine sulfate (HCQ) were purchased from Macklin (China). The antibody against cleaved Caspase-9 (#9508), Caspase-3 (#14220), PARP (#5625), p-p38 (#4511), p38 (#8690), p-ERK 1/2 (#4370), ERK 1/2 (#4695), p-JNK (#4668), JNK (#9252), p-AKT (#4060), AKT (#4691), p-PDK1 (#3438), p-PTEN (#9551), Cyclin E1 (#20808), PCNA (#2586) were obtained from Cell Signaling Technology (USA). The antibody against p-Chk1 (#HA721189), Cyclin A2 (#ET1612-26) were purchased from HUABIO (China). The antibody against Bcl-2 (#12789-1-AP), Bax (#60267-1-Ig), CHOP (#15204-1-AP) were purchased from Proteintech (China). MCF-7, MDA-MB-231, MDA-MB-453, and MCF-10A cell lines were purchased from Procell (China) and cultured according to the supplier’s recommended protocols.

### 2.2 Cellular cytotoxicity assay

Cellular cytotoxicity was evaluated using the CCK-8 assay. 3,000–8,000 cells per well were plated in 96-well plates, and the cells were cultured overnight at 37 °C to allow full adhesion. Subsequently, culture medium with different concentrations of the drug was freshly prepared and added, followed by an additional 48-h incubation. Finally, CCK-8 reagent (10% of the total volume) was added to the medium, and the cells were incubated for 1–3 h before measuring the absorbance at 450 nm using a multimode microplate reader.

### 2.3 Clonogenic assay

600 cells per well were plated in 6-well plates. After the preliminary formation of cell colonies (5-7 cells), various concentrations of drug were added. The culture medium containing freshly prepared drugs was replaced every 3 days. The cells were then cultured for 8–12 days until colonies containing more than 50 cells formed. Finally, the colonies were fixed in ice-cold methanol for 20 min and subsequently stained with 0.5% crystal violet.

### 2.4 Wound healing assay

3 × 10^5^ MDA-MB-231 cells per well were plated in a 6-well plate and cultured at 37 °C. A 200 μL pipette tip was used to create linear scratches once the cell monolayer reached about 95% confluency, and the cells were carefully rinsed with PBS to remove detached debris. To minimize the influence of cell proliferation, the culture medium was changed to L-15 medium supplemented with 1% FBS and CIr2 at final concentrations of 0, 100, or 200 μM. After 24 h of treatment, cell migration was quantified using the following formula:

$$\text{Migration area }\left(\%\right)=\left[\text{empty area }\left(0\;h\right)-\text{empty area }\left(24\;h\right)\right]/\text{empty area }\left(0\;h\right)\times 100.$$

### 2.5 Transwell assay

3 × 10^4^ MDA-MB-231 cells were seeded into the upper chambers of Transwell inserts (Corning, USA) in serum-free medium containing the indicated drugs. To serve as a chemoattractant, 400 μL of medium containing 20% FBS was filled in the lower chamber. Following 24 h culture, cells that had not migrated and remained on the upper surface of the insert were carefully removed. Subsequently, the cells that had traversed to the lower surface of the membrane were fixed and stained with 0.5% crystal violet. For quantification, five randomly selected fields (×100 magnification) were analyzed using phase-contrast microscopy.

### 2.6 Intracellular localization study

2 × 10^4^ MDA-MB-231 and MCF-7 cells were plated in 4-well confocal dishes and cultured at 37 °C overnight. After exposure to 400 nM CIr2 for 12 h at 37 °C, the cells were washed with PBS, and afterwards stained with MitoTracker Red for 30 min under dark conditions. Finally, the samples were rinsed with PBS, images were obtained using an 810 Laser Scanning Confocal Microscopy System (Zeiss, Germany).

### 2.7 ROS level analysis

The fluorescent probe 2′,7′-dichlorodihydrofluorescein diacetate (DCFH-DA) was used to assess intracellular ROS accumulation. 3 × 10^5^ MDA-MB-231 cells per well were seeded in 6-well plates for 12 h at 37 °C, then cells were exposed to CIr2 for 24 h. Following PBS washing, cells were stained with DCFH-DA for 30 min at 37 °C under dark conditions. Then the cells were harvested and resuspended in PBS containing 0.5% FBS. Subsequently, the samples were examined using a Novo Sampler Q flow cytometer (Agilent Technologies, USA).

### 2.8 MMP change analysis

MMP alterations were evaluated using the MMP assay kit with TMRE. MDA-MB-231 cells were plated in 35 mm confocal dishes and cultured overnight at 37 °C. After treatment with various concentrations of CIr2 for 16 h, the cells were stained with TMRE for 30 min under dark conditions, then rinsed with incubation buffer, and promptly visualized using a confocal microscopy system.

### 2.9 ATP depletion assay

ATP levels in cells were evaluated using a firefly luciferase-based assay kit. MDA-MB-231 cells were plated at 3 × 10^5^ cells per well in 6-well plates and cultured overnight at 37 °C. After exposure to various concentrations of CIr2 for 24 h, the cells were lysed in lysis buffer. A multimode microplate reader was used to measure the luminescence intensity.

### 2.10 Cell cycle assay

Cell cycle assessment followed the manufacturer’s instructions. MDA-MB-231 cells were seeded in 6-well plates at 3 × 10^5^ cells per well and cultured overnight at 37 °C. After being exposed to different CIr2 concentrations for 24 h, the cells were collected and fixed for 6 h in 70% ice-cold ethanol. Following fixation, the cells were stained with a 20 μg/mL propidium iodide solution containing RNase under dark conditions for 30 min. The samples were then examined using flow cytometry.

### 2.11 Cell apoptosis assay

To assess how CIr2 affects the induction of apoptosis, 3 × 10^5^ MDA-MB-231 cells per well were plated in 6-well plates and cultured at 37 °C overnight. Following exposure to various concentrations of CIr2 or cisplatin for 24 h, the cells were collected, rinsed with PBS, and stained with Annexin V-FITC solution under dark conditions for 30 min. The samples were then analyzed using flow cytometry.

### 2.12 Analysis of mitochondrial morphology

MDA-MB-231 cells (3 × 10^6^ cells) were exposed to 500 nM CIr2 at 37 °C for 24 h. After treatment, cells were pre-fixed with 2.5% glutaraldehyde at 4 °C for 2 h, then washed with PBS, and post-fixed with 1% osmium tetroxide at 4 °C for 1 h. Following a wash with cold PBS, the cells underwent a dehydration process using a graded series of ethanol followed by acetone to ensure complete removal of water. They were then embedded in PON 812 resin (SPI Supplies, USA) at 65 °C for 72 h. The embedded samples were sectioned, stained with lead citrate, and subsequently analyzed using a transmission electron microscope (TEM) instrument (HT7700, Japan).

### 2.13 Western blot analysis

3 × 10^5^ MDA-MB-231 and MDA-MB-453 cells per well were plated in 6-well plates and cultured at 37 °C overnight. Following treatment with CIr2 and various inhibitors at the indicated concentrations and time points, for protein extraction, cells were lysed with RIPA lysis buffer containing protease inhibitors. Protein lysates were resolved by electrophoresis on 4%–20% SDS–PAGE gels (Beyotime, China) and subsequently transferred onto PVDF membranes. To block non-specific antibody binding, membranes were incubated with 5% skim milk for 1 h at room temperature. After blocking, they were exposed to primary antibodies at 4 °C overnight. Following several washes with TBST buffer, membranes were treated with HRP-linked secondary antibodies for 1 h. Protein bands were visualized using a ChemiDoc imaging system (Bio-Rad, USA).

### 2.14 RNA sequence analysis

3 × 10^5^ MDA-MB-231 cells per well were plated in 6-well plates and cultured at 37 °C overnight. Subsequently, cells were exposed to 500 nM CIr2 for another 24 h. Following ice-cold PBS washing, cells were lysed using Trizol reagent (Invitrogen, USA) to extract total RNA. The quality of RNA was evaluated by a NanoDrop spectrophotometer (Thermo Scientific, USA). RNA sequencing libraries were prepared and analyzed using an Illumina platform (Wuhan Metware Biotech Co., Ltd., China).

### 2.15 *In vivo* antitumor activity study

All animal experiments were performed with the approval of the Ethics Committee of Guilin Medical University (protocol number GLMC-IACUC-20243159), following the institutional guidelines for animal care and use.

#### 2.15.1 Preparation of pluronic F127 hydrogels encapsulated with CIr2 (CIr@F127)

Pluronic F-127 (F127) is an amphiphilic A-B-A type triblock copolymer widely used in drug delivery. We prepared a drug hydrogel, CIr@F127, containing 21% F127 and 8 mg/L CIr2 for the subsequent xenograft study. The detailed preparation process, degradation analysis, and pharmacokinetic study of CIr@F127 were reported in our previous research (Lin et al., 2024).

#### 2.15.2 Xenograft studies

Female BALB/c nude mice (6 weeks old, weighing approximately 20 g) were purchased from Hunan SJA Laboratory Animal (China). A total of 4 × 10^6^ MDA-MB-231 cells were suspended in 200 µL of serum-free medium, combined with an equal volume of Matrigel (ABW Bio, China), and injected subcutaneously into the fourth mammary fat pad of the mice. Tumor size and body weight were measured twice a week, and formula V = (length) × (width)^2^/2 was used to determine the tumor volume (V). The mice were divided into two groups at random (n = 7/group) once the tumors had grown to an average volume of 100 mm^3^ (approximately 5 weeks post-inoculation). The groups received intratumoral injections of F127 (vehicle control) or CIr@F127 (equivalent to 8 mg/kg free CIr2) every 5 days. Following a 4-week treatment period, the animals were sacrificed, and tumor tissues were harvested for histological examination, while TUNEL staining was also performed to evaluate apoptosis in tumor cells.

### 2.16 Statistical analysis

All cell culture experiments were performed with at least three independent biological replicates. Data are expressed as the mean ± standard error of the mean (SEM). The significance of differences between two groups was evaluated using Student’s t-test. *P* values <0.05 were considered statistically significant.

## 3 Results and discussion

### 3.1 Chemistry

The chemical structure of CIr2 was shown in Supplementary Figure S1. The detailed syntheses and characterization process were described in our previous study (Lin et al., 2024).

### 3.2 CIr2 selectively inhibits proliferation and migration of TNBC cells

Numerous iridium-based complexes have demonstrated notable cytotoxic effects against cancer cells. In the present study, we assessed the cytotoxicity of CIr2 across various breast cancer cell lines, including MCF-7 (Luminal A), MDA-MB-231 (TNBC), MDA-MB-453 (TNBC), and the normal breast epithelial cell line MCF 10A. Cisplatin served as the positive control. Table 1 presents the IC_50_ values measured following 48 h of drug exposure.

**TABLE 1:** Summary of the 48-h IC_50_ (μM) values obtained for the compounds in different cell lines.

Compounds	MCF-7	MDA-MB-231	MDA-MB-453	MCF 10A
CIr2	1.06 ± 0.19	0.15 ± 0.01	0.22 ± 0.01	0.88 ± 0.06
Cisplatin	11.94 ± 2.24	>50	26.50 ± 4.42	6.33 ± 0.59

As shown in Table 1, CIr2 exhibited potent anti-proliferative activity across all examined cell lines. Analysis of the IC_50_ values revealed that CIr2 selectively inhibits TNBC cell lines, showing micromolar potency in MDA-MB-231 (0.15 ± 0.01 μM) and MDA-MB-453 (0.22 ± 0.01 μM) in contrast to the normal breast cell line MCF 10A (0.88 ± 0.06 μM). Notably, CIr2 exhibited markedly stronger cytotoxic effects toward TNBC cell lines compared to cisplatin, with cisplatin IC_50_ values exceeding 50 μM in MDA-MB-231 and 26.50 ± 4.42 μM in MDA-MB-453, respectively. Since cisplatin primarily exerts its anticancer effects by inducing DNA damage and activating apoptotic signaling pathways, these findings indicate that CIr2 employs alternative mechanisms to suppress TNBC cell growth. MDA-MB-231 was chosen for mechanistic studies of CIr2 in TNBC due to its cytotoxic profile.

**FIGURE 1:**
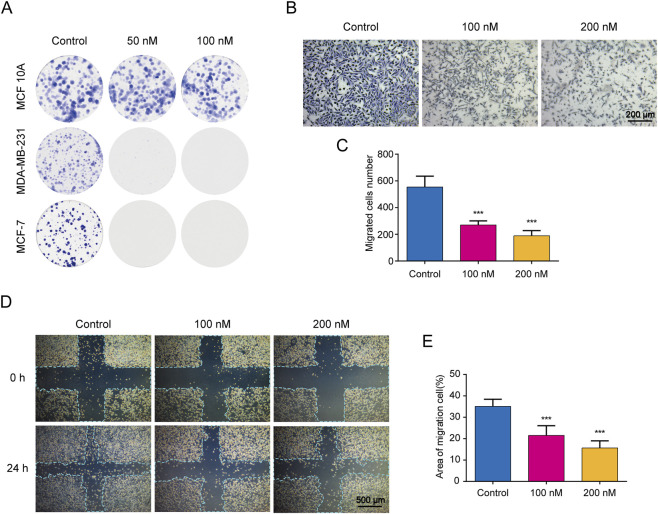
Antiproliferative and anti-migratory effects of CIr2 on TNBC cells. **(A)** CIr2 inhibited colony formation in the indicated cell lines. **(B)** Transwell analysis of MDA-MB-231 cells migration and invasion. Scale bar: 200 μm. **(C)** Quantification of migrated cells in the Transwell assay. **(D)** Wound healing analysis of MDA-MB-231 cells migration. Scale bar: 500 μm. **(E)** Quantification of cell migration in the wound healing assay. (***p < 0.001).

Additionally, we conducted colony formation assays in MDA-MB-231, MCF-7, and MCF 10A cells to further assess the inhibitory effects on cell proliferation of CIr2. The MDA-MB-453 cell line was excluded due to its semi-adherent nature, making it unsuitable for this assay. Exposure of MDA-MB-231 and MCF-7 cells to 50 nM CIr2 significantly reduced colony formation, and complete inhibition was observed at 100 nM. In contrast, CIr2 exhibited no significant cytotoxicity towards colony-forming ability of MCF 10A cells even at 100 nM (Figure 1A).

The highly aggressive migratory capacity of TNBC to distant organs makes metastasis a persistent challenge in its clinical treatment. Wound healing and Transwell assays were performed to evaluate the effects of CIr2 on the migration and invasion of MDA-MB-231 cells. As illustrated in Figures 1B–E, CIr2 dose-dependently inhibited the migratory and invasive abilities of the cells in contrast to the control. Notably, at lower concentrations (50–100 nM), CIr2 exhibited a suppressive trend on cell migration and invasion without significantly affecting cell viability after 24 h treatment, suggesting that the observed anti-migratory effects are not solely attributable to acute cytotoxicity (Supplementary Figures S2A-E). Taken together, all of the results indicate that CIr2 possesses promising antitumor activity, particularly against TNBC cells, and holds potential for further development as an anti-TNBC drug.

### 3.3 CIr2 targets mitochondria and disrupts mitochondrial function

#### 3.3.1 CIr2 localizes to mitochondria

**FIGURE 2:**
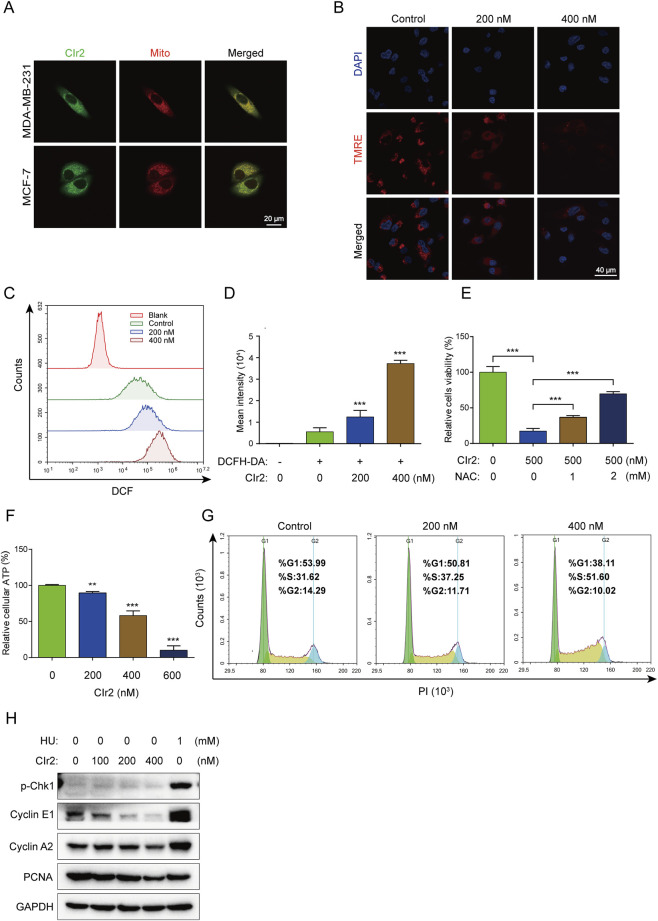
CIr2 triggers mitochondrial dysfunction and induces S phase cell cycle arrest. **(A)** Confocal imaging was performed on MDA-MB-231 cells treated with CIr2 (green) for 12 h and stained with a mitochondrial marker (red); scale bar: 20 μm. **(B)** Confocal imaging of TMRE levels following a 16 h CIr2 treatment of MDA-MB-231 cells; scale bar: 40 μm. **(C,D)** Quantification of intracellular ROS levels by flow cytometry following a 24 h CIr2 treatment of MDA-MB-231 cells. **(E)** Viability of MDA-MB-231 cells treated with NAC and CIr2 (500 nM) for 24 h for ROS scavenging analysis. **(F)** ATP levels in MDA-MB-231 cells after CIr2 treatment for 24 h. **(G)** Cell cycle analysis of MDA-MB-231 cells treated with CIr2 for 24 h. **(H)** Western blot analysis of indicated proteins in MDA-MB-231 cells following 24 h CIr2 or HU treatment. (**p < 0.01, ***p < 0.001).

Research on the subcellular localization of phosphorescent metal complexes offers valuable insights into their anticancer mechanisms (Tan et al., 2021). In our previous research, we identified mitochondria as the primary target of CIr2 in human pancreatic cancer cells. Given the variability in organelle localization of anticancer complexes across different cancer cell types (Marullo et al., 2013) and the pronounced heterogeneity of TNBC cells, we investigated the subcellular localization of CIr2 in MDA-MB-231 and MCF-7 cells using co-localization staining with MitoTracker Red. As illustrated in Figure 2A, CIr2-derived green fluorescence strongly overlaps with the red mitochondrial signals, indicating that CIr2 predominantly accumulates in the mitochondria of these breast cancer cells.

#### 3.3.2 CIr2 induces mitochondrial membrane potential loss

Given that CIr2 accumulates in the mitochondria, its impact on mitochondrial function was further examined. MMP reflects the voltage gradient across the inner mitochondrial membrane, serving as a vital parameter for evaluating mitochondrial integrity and functional status (Zorova et al., 2018). To investigate whether CIr2-induced cell death is linked to mitochondrial damage, MMP was assessed using TMRE, a dye that accumulates selectively in mitochondria depending on membrane potential (Galluzzi et al., 2007). The red fluorescence of TMRE was visualized using confocal microscopy. Figure 2B illustrates that CIr2 at 200 nM and 400 nM induced a marked, dose-dependent decline of MMP in contrast to the control. These findings suggest that CIr2 induces mitochondrial dysfunction as a central mechanism of action.

#### 3.3.3 CIr2 increases intracellular ROS production

Intracellular ROS are predominantly generated by mitochondria, and excessive ROS levels can cause mitochondrial damage. Numerous studies have demonstrated that iridium complexes can trigger various forms of cancer cell death through ROS generation (Chen J. et al., 2023; Zhou et al., 2024; Guan et al., 2021). In this study, flow cytometry with the ROS-sensitive probe DCFH-DA was employed to measure intracellular ROS production. Following 24 h CIr2 treatment, MDA-MB-231 cells exhibited a significant, dose-dependent increase in DCF fluorescence intensity, indicating enhanced ROS production. Cells treated with 400 nM CIr2 showed a roughly 6.5-fold increase in DCF fluorescence intensity (Figures 2C,D). Confocal microscopy further confirmed this dose-dependent rise in ROS levels in CIr2-treated MDA-MB-231 cells (Supplementary Figure S3). To elucidate the contribution of ROS in CIr2-induced cell death, we examined how ROS inhibition affects its therapeutic efficacy. NAC was used as a ROS scavenger that demonstrated a dose-dependent increase in cell viability (Figure 2E). These findings indicate that ROS generation is crucial for CIr2-induced cell death.

#### 3.3.4 CIr2 depletes intracellular ATP levels

Since mitochondria are the primary organelles of ATP synthesis, we assessed the intracellular ATP levels in MDA-MB-231 cells following CIr2 treatment. CIr2 exposure caused a dose-dependent decline in ATP levels, with a 600 nM concentration causing a 90% decrease compared to untreated cells (Figure 2F). Collectively, these results confirm that CIr2 induces mitochondrial dysfunction, leading to excessive ROS production and ATP depletion.

#### 3.3.5 CIr2 induces S-phase cell cycle arrest

Several studies have demonstrated that iridium (III) complexes suppress cancer cell growth through cell cycle arrest (Hao et al., 2021; Chen L. et al., 2023). Therefore, we evaluated the effect of CIr2 on cell cycle progression by flow cytometric analysis. A dose-dependent increase in the proportion of MDA-MB-231 cells in the S phase was observed after CIr2 exposure, while the percentages of cells in the G0/G1 and G2/M phases reduced relative to the control group (Figure 2G). This suggests that CIr2 significantly inhibits cell growth in the S phase of the cell division. To further evaluate the expression levels of S phase-related proteins following CIr2 treatment, Western blotting was performed, with hydroxyurea (HU), a classic S phase arrest inducer, serving as the positive control. Our results revealed that, unlike HU, CIr2 treatment did not induce the phosphorylation of Chk1 at Ser345. Instead, it was accompanied by the downregulation of Cyclin E1, Cyclin A2, and PCNA (Figure 2H). This suggests that CIr2-induced S phase arrest is independent of the canonical ATR-Chk1 DNA damage checkpoint pathway. Given that DNA synthesis during the S phase imposes a high energy demand, and considering our observation of CIr2-induced mitochondrial dysfunction and ATP depletion, we propose that CIr2 triggers a passive S phase arrest resulting from metabolic and biosynthetic impairment, rather than the active checkpoint-mediated arrest observed with HU. Similar findings linking drug-induced mitochondrial dysfunction to S phase arrest and cyclin downregulation have been reported in previous studies (Hung et al., 2013; Yang et al., 2016).

### 3.4 CIr2 induces caspase-independent cell death

**FIGURE 3:**
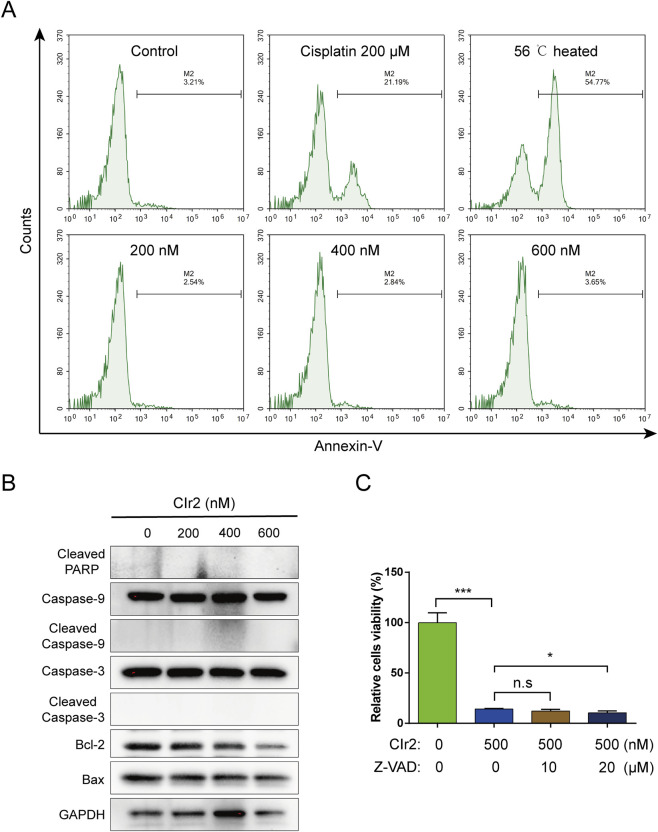
Apoptosis detection in TNBC cells treated with CIr2. **(A)** Annexin V-based flow cytometry of MDA-MB-231 cells after 24 h treatment with CIr2 at various concentrations. Positive controls were cells exposed to 56 °C for 20 min or treated with 200 μM cisplatin for 24 h. **(B)** Western blot analysis of indicated proteins in MDA-MB-231 cells following 24 h CIr2 treatment at various concentrations. **(C)** MDA-MB-231 cell viability after Z-VAD-FMK and CIr2 treatment. (*p < 0.05, ***p < 0.001).

The potent anticancer activity of CIr2 led us to explore its fundamental mechanisms. In our previous study, CIr2 was demonstrated to trigger ceramide redistribution and induce apoptosis in pancreatic cancer cells (Lin et al., 2024). To investigate the apoptotic effect of CIr2 on MDA-MB-231 cells, we performed Annexin V labeling assays to detect phosphatidylserine externalization, an early marker of apoptosis (Balasubramanian et al., 2007). Surprisingly, CIr2 treatment caused only a minimal increase in Annexin V-positive cells compared to the cisplatin- and heat-treated positive controls (Figure 3A), indicating that the cell membrane integrity remained largely intact, even at concentrations exceeding twice the IC_50_. Additionally, since caspase activation is a hallmark of apoptosis (Riedl and Shi, 2004), Western blot analysis was employed to examine the expression of apoptosis-related proteins, including cleaved PARP, Caspase-9, Caspase-3, Bcl-2, and Bax, in MDA-MB-231 cells following CIr2 treatment. As shown in Figure 3B, CIr2 did not activate Caspase-9, Caspase-3 or PARP, nor did it significantly affect the levels of Bcl-2 and Bax. A similar trend was found in the MDA-MB-453 TNBC cells. Conversely, cells exposed to staurosporine, an established apoptosis inducer, showed clear PARP cleavage (Supplementary Figure S4). Furthermore, treatment with Z-VAD-FMK, a pan-caspase inhibitor, failed to reduce the cytotoxicity of CIr2 (Figure 3C). The absence of caspase activation and the ineffectiveness of Z-VAD-FMK indicate that CIr2 acts independently of the canonical apoptotic machinery. This distinguishes CIr2 from conventional metal-based chemotherapeutics (Wu et al., 2024; Guo et al., 2025; Pang et al., 2025), suggesting that the compound engages unique signaling networks to bypass apoptosis resistance frequently observed in TNBC cells.

### 3.5 CIr2 induces paraptotic cell death accompanied by ER stress and p38 MAPK activation

#### 3.5.1 Transcriptomic profiling reveals ER stress and MAPK pathway activation upon CIr2 treatment

**FIGURE 4:**
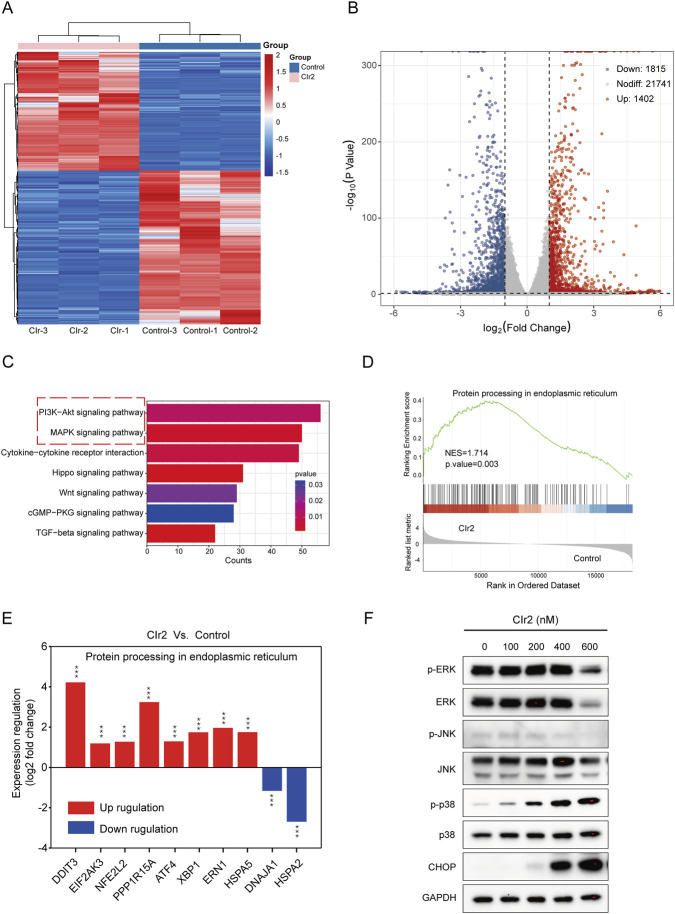
Assessment of critical pathways and biological processes linked to CIr2 sensitivity in MDA-MB-231 cells. **(A)** Heatmap of differentially expressed genes between CIr2-treated and control cells, identified by RNA sequencing. **(B)** Volcano plot. **(C)** KEGG pathway analyses. **(D)** GSEA analysis showed enrichment of the protein processing in the endoplasmic reticulum pathway gene set. NES: normalized enrichment score. **(E)** Alterations in the expression of ER stress-associated genes linked to the protein processing in the endoplasmic reticulum pathway. **(F)** Western blot analysis of the indicated protein in MDA-MB-231 cells treated with various concentrations of CIr2 for 24 h (***p < 0.001).

RNA-seq analysis was performed on MDA-MB-231 cells following 24 h treatment with 500 nM CIr2 to explore its therapeutic mechanism in inducing TNBC cell death. As shown in Figures 4A,B, the treatment caused significant changes in transcriptional profiles, with 1,402 genes upregulated and 1,815 genes downregulated compared with the control group. KEGG pathway analysis revealed significant alterations in various key cancer-related pathways, with the MAPK and PI3K-AKT signaling pathways playing prominent roles in the response to CIr2 treatment (Figure 4C and Supplementary Figure S5). Furthermore, Gene Set Enrichment Analysis (GSEA) showed that genes involved in protein processing in the endoplasmic reticulum pathway were substantially enriched in MDA-MB-231 cells after CIr2 treatment (Figures 4D,E). Notably, several genes closely associated with ER stress were substantially dysregulated (Supplementary Figure S6), with DDIT3 exhibiting the most prominent upregulation. The CHOP protein encoded by DDIT3 is a well-established marker of ER stress (Oyadomari and Mori, 2004; Nishitoh, 2012), suggesting that CIr2 induces pronounced ER stress in MDA-MB-231 cells.

The PI3K-AKT and MAPK signaling pathways are essential for regulating fundamental cellular functions (Martini et al., 2014; Kim and Choi, 2010). To investigate whether these pathways are involved in CIr2-mediated cytotoxicity, we assessed the activation of key signaling proteins in MDA-MB-231 cells following CIr2 treatment. p38 phosphorylation was elevated in a dose-dependent manner, whereas JNK phosphorylation was not activated following CIr2 treatment (Figure 4F; Supplementary Figure S7). Interestingly, the basal levels of both phosphorylated and total ERK were initially high, but only significantly decreased at high concentrations of CIr2 (600 nM). Evidence increasingly points to a potential role of strong ERK activation in promoting apoptosis resistance in TNBC cells (Bartholomeusz et al., 2012; Salaroglio et al., 2019; Paramanantham et al., 2021). In contrast, the PI3K-AKT pathway demonstrated no apparent activation or inhibition tendency following CIr2 treatment in MDA-MB-231 cells (Supplementary Figure S8). Consistent with the transcriptomic findings, Western blot analysis further confirmed a pronounced, dose-dependent increase in CHOP protein expression (Figure 4F), validating the induction of ER stress by CIr2.

#### 3.5.2 CIr2 induces paraptosis-like morphological and ultrastructural features

Paraptosis is a non-apoptotic cell death critically influenced by activation of the MAPK pathway (Che et al., 2024). ER stress is also recognized as a hallmark of paraptosis (Hanson et al., 2023), and the CHOP protein has been reported to promote paraptosis under prolonged ER stress (Yoon et al., 2014; Zhang et al., 2020). The concurrent activation of p38 MAPK, induction of ER stress, and the non-apoptotic nature of CIr2-induced cytotoxicity prompted us to further investigate whether CIr2 induces paraptosis-like cell death.

**FIGURE 5:**
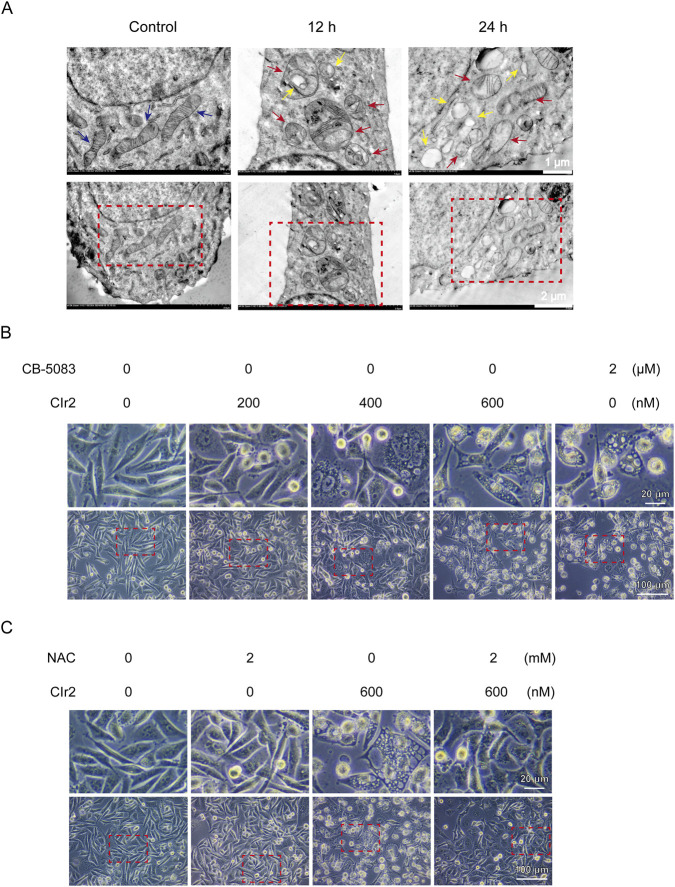
Morphological and ultrastructural analyses of CIr2-induced paraptosis in TNBC cells. **(A)** Mitochondrial ultrastructural alterations of MDA-MB-231 cells following 500 nM CIr2 treatment were examined by TEM, with control, 12 h, and 24 h groups processed in parallel. Blue arrows denote healthy mitochondria, red arrows denote swollen mitochondria, and yellow arrows denote vacuoles. Scale bar: 1 μm and 2 μm. **(B)** MDA-MB-231 cells were treated with the indicated concentrations of CIr2 or CB-5083 for 24 h, images were obtained using phase-contrast microscope. Scale bar: 20 μm and 100 μm. **(C)** MDA-MB-231 cells were treated with the indicated concentrations of CIr2 or NAC for 24 h, images were obtained using phase-contrast microscope. Scale bar: 20 μm and 100 μm.

In line with current high-impact studies, paraptosis has primarily been characterized based on *in vitro* morphological and ultrastructural features, together with the exclusion of other regulated cell death pathways (Wang et al., 2024; Dai et al., 2025; Tamez-Fernández et al., 2025). Transmission electron microscopy (TEM) was employed to assess the ultrastructural alterations in MDA-MB-231 cells after CIr2 treatment. As shown in Figure 5A, the control cells exhibited intact mitochondria with well-defined double membranes, smooth outer membranes, and clearly visible inner membrane folds. In contrast, most mitochondria displayed disrupted cristae and signs of swelling following 12 h of CIr2 treatment. With prolonged treatment (24 h), mitochondrial damage became more pronounced, leading to extensive vacuole formation. Comparable ultrastructural changes (mitochondrial swelling, vacuolation, and ER dilation) have been reported for ASb-2 peptide–iridium hybrids as well as for Ir1 in HepG2 cells, both of which implicate mitochondrial–ER stress cross-talk in mediating the paraptotic phenotype (Balachandran et al., 2021).

To further substantiate these findings, light microscopy–based morphological analyses were performed, with CB-5083—a well-established paraptosis inducer in MDA-MB-231 cells (Lee et al., 2024)—used as a positive control. As shown in Figure 5B, CIr2 treatment resulted in a dose-dependent increase in detached, non-adherent cells accompanied by marked morphological alterations, including cell swelling and extensive cytoplasmic vacuolization. These features closely resembled those observed following CB-5083 treatment, supporting the association of CIr2-induced cytotoxicity with paraptotic cell death. Similar morphological changes were also observed in MCF-7 breast cancer cells (Supplementary Figure S9A). Importantly, CIr2-induced vacuoles were surrounded by single membranes, distinguishing them from the double-membrane autophagosomes characteristic of autophagy (Yu et al., 2018). Together, these morphological and ultrastructural features strongly indicate that CIr2 induces paraptosis-like cell death in breast cancer cells.

#### 3.5.3 ROS-mediated mitochondrial dysfunction initiates CIr2-induced paraptosis

To exclude the involvement of other regulated non-apoptotic cell death pathways, including ferroptosis (Guo et al., 2022), necroptosis, and autophagy, MDA-MB-231 cells were treated with the corresponding inhibitors Ferrostatin-1, Necrostatin-1, and HCQ. None of these inhibitors significantly rescued CIr2-induced cytotoxicity (Supplementary Figures S10A–C). In addition, pharmacological inhibition of p38 MAPK using SB202190 or suppression of ER stress with 4-PBA failed to reverse CIr2-induced cell death (Supplementary Figures S10D, E), indicating that neither p38 MAPK activation nor ER stress alone is essential for the execution of CIr2-induced paraptosis.

In contrast, treatment with the ROS scavenger NAC effectively rescued CIr2-induced cytotoxicity. Consistent with this functional rescue, NAC markedly attenuated CIr2-induced cytoplasmic vacuolization in both MDA-MB-231 (Figure 5C) and MCF-7 cells (Supplementary Figure S9B). These findings demonstrate that ROS accumulation and mitochondrial dysfunction represent initiating events in CIr2-induced paraptosis, whereas ER stress and p38 MAPK activation function as downstream stress responses rather than essential execution mechanisms. Interestingly, a recent study reported that Flemiphilippinin A induces paraptosis in lung cancer cells primarily through endoplasmic reticulum stress and CHOP-mediated mitochondrial dysfunction. In that study, pharmacological suppression of ER stress using 4-PBA effectively reversed Flemiphilippinin A–induced cell death, whereas ROS scavenging with NAC failed to confer protection (Xu et al., 2025)^.^ This mechanistic pattern contrasts with our findings, in which NAC, but not ER stress or p38 MAPK inhibition, effectively rescued CIr2-induced paraptosis. Together, these observations suggest that functional crosstalk between mitochondria and the endoplasmic reticulum is a common feature of paraptosis, while the primary initiating signal may vary depending on the nature of the stimulus. Specifically, paraptosis may be initiated either by mitochondrial dysfunction–driven oxidative stress or by ER stress–dominated signaling, ultimately converging on a shared paraptotic phenotype.

Although our findings demonstrate a clear link between mitochondrial dysfunction, ER stress, and p38 activation, the precise molecular targets of CIr2 remain to be identified. It is possible that CIr2 interacts with specific mitochondrial or ER-associated proteins to initiate paraptosis-like events. Future studies employing proteomic or chemoproteomic profiling will be essential to pinpoint the direct targets and upstream regulators of CIr2. Notably, similar strategies have been successfully utilized to elucidate the targets of other iridium-based compounds (Li et al., 2025). Such target identification would provide valuable insights into the unique mechanism of CIr2 and guide the rational design of more selective analogs for refractory cancers.

### 3.6 CIr2 suppresses TNBC tumor growth *in vivo* with minimal toxicity

**FIGURE 6:**
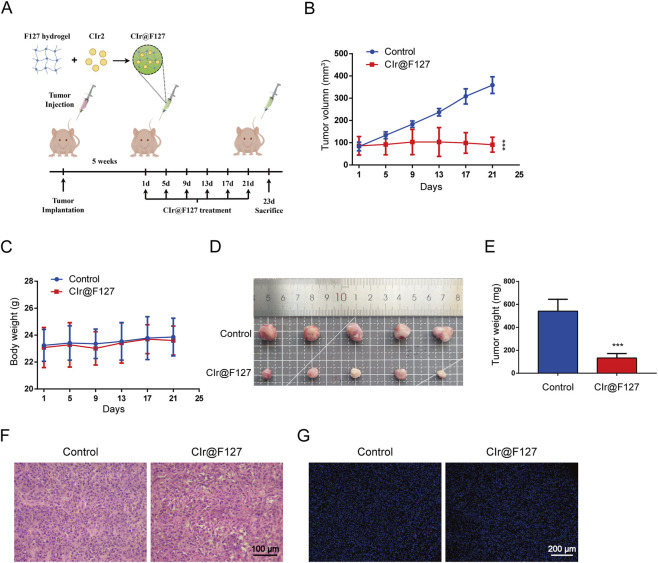
Anticancer effect of CIr2 *in vivo*. **(A)** Schematic representation of the xenograft model and CIr2 treatment protocol. The figure was generated using Figdraw. **(B)** The tumor volume growth curves in the vehicle and CIr@F127 (equivalent to 8 mg/kg free CIr2) treatment group. **(C)** Changes in body weight of mice in each group throughout the study. **(D)** Photographs of tumors in each group. **(E)** Tumor weights recorded after euthanizing the mice. **(F)** Tumor section images from each group after H&E staining are shown. Scale bar: 100 μm. **(G)** Tumor section images from each group following TUNEL staining. Scale bar: 200 μm. (***p < 0.001).

Given the potent *in vitro* activity of CIr2, its anticancer activity was assessed *in vivo* in nude mice bearing MDA-MB-231 tumors. As shown in Figures 6A–E, following 25 days of treatment, tumor volume in the control group grew sharply, while tumor growth in the CIr@F127-treated group was markedly suppressed, with a tumor inhibition rate of 75.4%. Importantly, treatment with CIr@F127 was well tolerated, with no mortality or notable changes in body weight observed over the course of the study. Furthermore, our previous study demonstrated that CIr@F127 treatment at the same dose (8 mg/kg) did not significantly affect the major organs of mice (Lin et al., 2024), indicating minimal severe side effects under these conditions. In addition, immunohistochemical analysis was conducted to evaluate the antitumor effects of CIr@F127 treatment. HE staining demonstrated that tumors in the control group exhibited a high density of cell nuclei, closely packed intercellular spaces, and relatively preserved cellular morphology (Figure 6F). Conversely, the CIr@F127-treated group displayed a decreased nuclear density along with enlarged and irregular intercellular spaces, suggesting that CIr2 disrupts tumor tissue architecture and exerts anticancer effects. However, as shown in Figure 6G, TUNEL staining indicated minimal difference in the red fluorescence marking apoptotic cells between the control and CIr@F127-treated groups, with only a few cells showing TUNEL positivity. It should be noted that, unlike apoptosis, paraptosis currently lacks validated molecular markers for direct identification *in vivo* and is primarily defined by distinctive morphology features, which are most reliably assessed by transmission electron microscopy. Consequently, most studies characterize paraptosis based on comprehensive *in vitro* morphological and mechanistic evidence, while *in vivo* experiments mainly focus on antitumor efficacy and exclusion of apoptosis. In this context, our *in vivo* analyses were designed to evaluate therapeutic efficacy and biosafety, whereas the paraptotic nature of CIr2-induced cell death was rigorously established *in vitro*. Future studies incorporating ultrastructural analyses of tumor tissues will be important to further elucidate paraptosis under physiological conditions. Collectively, these *in vivo* results reinforce the translational promise of paraptosis-inducing iridium complexes, consistent with previous reports that IrM2 effectively suppressed tumor growth in animal models without overt toxicity (He et al., 2018).

### 3.7 Proposed mechanism of CIr2-induced paraptosis in TNBC cells

**FIGURE 7:**
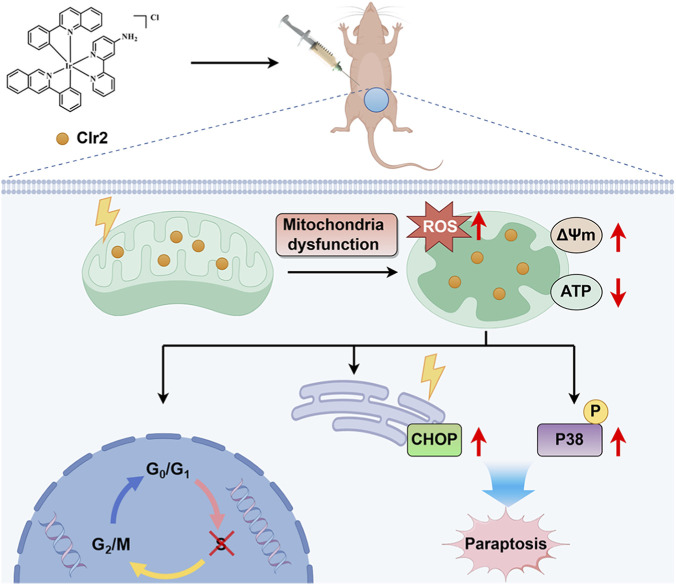
Proposed mechanism of CIr2-induced paraptosis in TNBC cells. CIr2 triggers mitochondrial dysfunction and ER stress, leading to caspase-independent paraptotic cell death and tumor growth suppression. The figure was generated using Figdraw.

Collectively, our findings delineate a non-apoptotic mechanism by which CIr2 induces paraptosis in TNBC cells (Figure 7). CIr2 accumulation in mitochondria causes mitochondrial dysfunction, oxidative stress, and ATP depletion, which in turn activate ER stress and p38 MAPK signaling. The resulting mitochondrial–ER crosstalk leads to extensive cytoplasmic vacuolation and caspase-independent paraptotic cell death. These results suggest that CIr2 holds therapeutic potential as a paraptosis-inducing agent for triple-negative breast cancer.

## 4 Conclusion

In summary, our research demonstrates that the novel cyclometalated iridium (III) complex CIr2 shows potent and selective cytotoxicity toward triple-negative breast cancer cells. CIr2 localizes to mitochondria, where it induces membrane potential loss, excessive ROS generation, and ATP depletion, leading to mitochondrial dysfunction. These mitochondrial perturbations trigger ER stress and are accompanied by activation of the p38 MAPK signaling pathway, collectively contributing to caspase-independent paraptotic cell death. Morphological and transcriptomic analyses further support that CIr2 induces typical paraptotic features, including mitochondrial swelling and ER dysfunction as well as cytoplasmic vacuolation. Furthermore, CIr2 significantly suppresses TNBC xenograft growth *in vivo* with minimal systemic toxicity. Collectively, these findings reveal a novel pharmacological mechanism whereby a cyclometalated Ir(III) complex induces paraptosis through mitochondrial–ER crosstalk. CIr2 thus emerges as a promising therapeutic candidate for developing anticancer agents that can overcome apoptosis resistance in TNBC and possibly other refractory tumors. Future studies focusing on the molecular targets of CIr2 may further enhance its therapeutic potential.

## Data Availability

The original contributions presented in the study are publicly available. This data can be found here: GEO repository, accession number GSE316771.
