# Supplementary material for: A cyclometalated iridium(III) complex induces paraptotic cell death via mitochondrial dysfunction and ER stress in triple-negative breast cancer cells
Source: Front Pharmacol. 2026 Jan 26;17:1739226. doi: 10.3389/fphar.2026.1739226 (PMC12883731; doi:10.3389/fphar.2026.1739226)
Supplement: Supplementary file 1 [file Supplementaryfile1.docx]

Supplementary Material


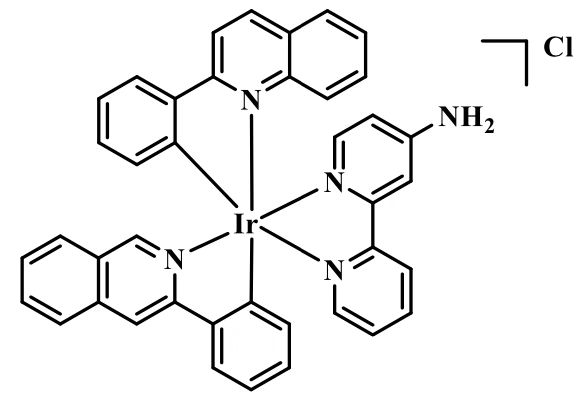


**Fig. S1.** Chemical structure of CIr2.


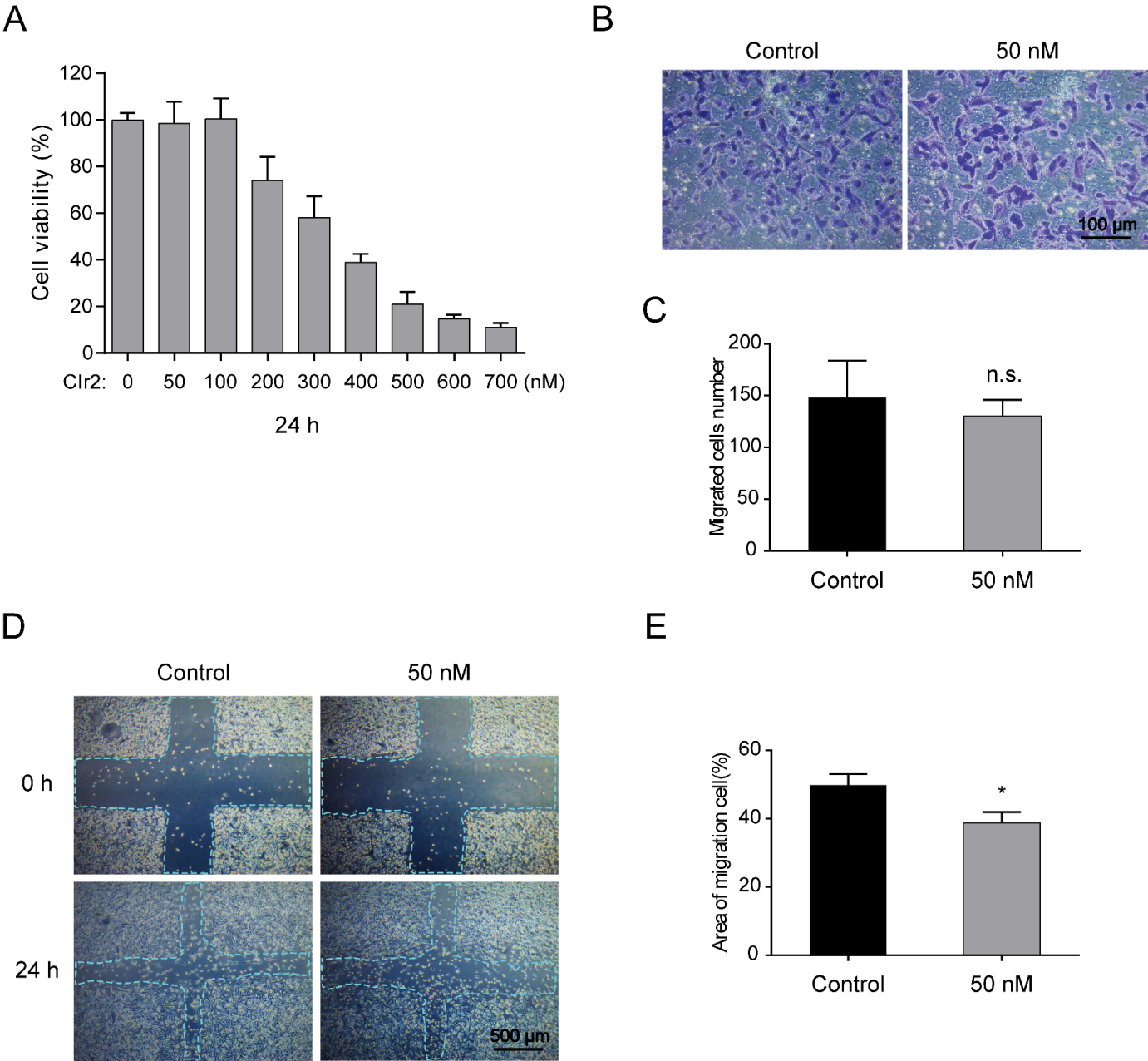


**Fig. S2.**  (A) MDA-M-231 cells were treated with the indicated concentrations of CIr2 for 24 h, and cell viability was evaluated using the CCK-8 assay. (B) Transwell analysis of MDA-MB-231 cells migration and invasion. Scale bar: 100 μm. (C) Quantification of migrated cells in the transwell assay. (D) Wound healing analysis of MDA-MB-231 cells migration. Scale bar: 500 μm. (E) Quantification of cell migration in the wound healing assay. ( **p* < 0.05)


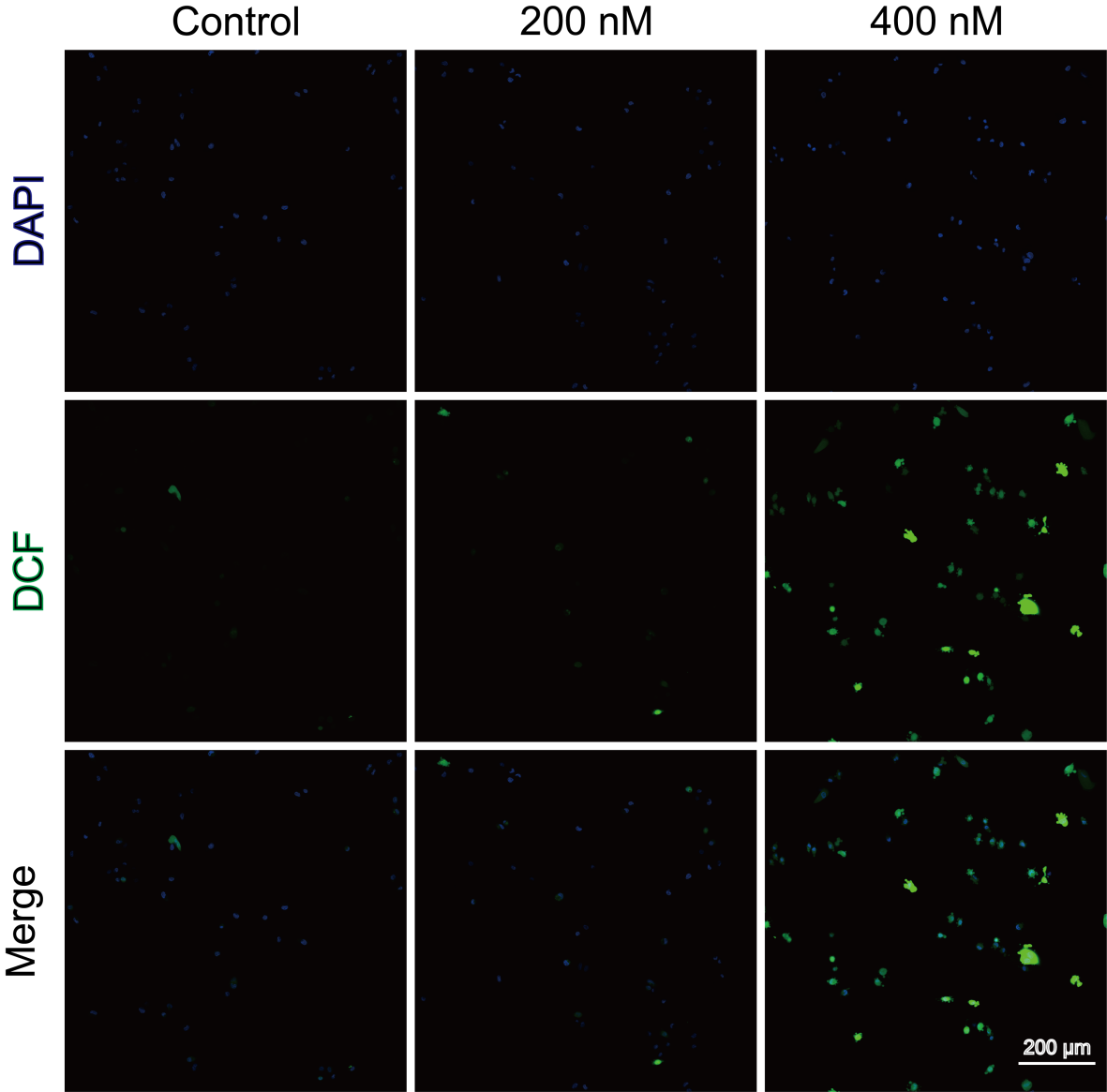


**Fig. S3.** Confocal microscopy analysis of DCF levels after MDA-MB-231 cells were treated with CIr2 for 24 h; scale bar: 200 μm.


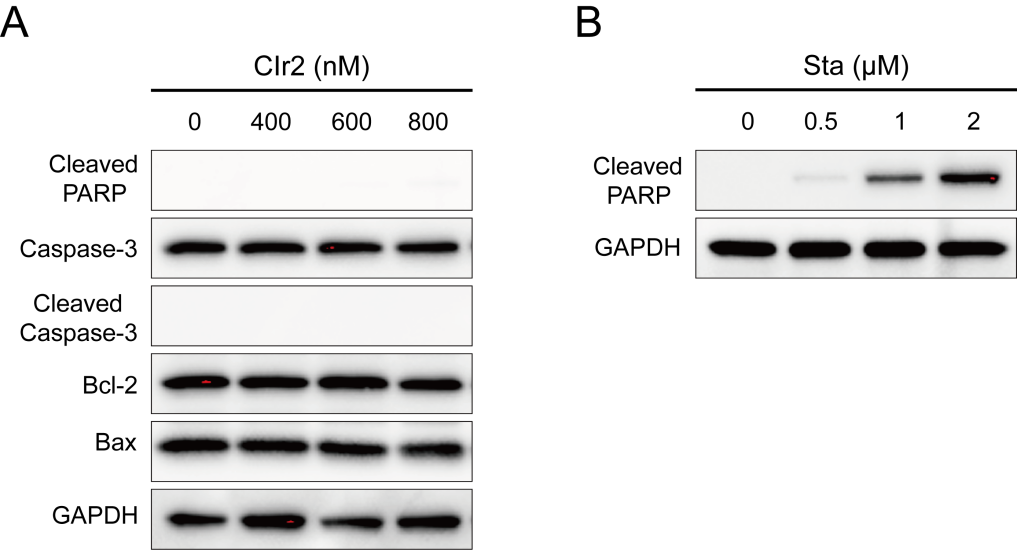


**Fig. S4.** (A) Western blot analysis of cleaved PARP, casepase-3, bcl-2 and bax after treatment of MDA-MB-453 cells with CIr2 at 400, 600, 800 nM respectively for 24h. (B) Western blot analysis of cleaved PARP after treatment of MDA-MB-453 cells with Staurosporine at 0.5, 1, 2 μM respectively for 6h.


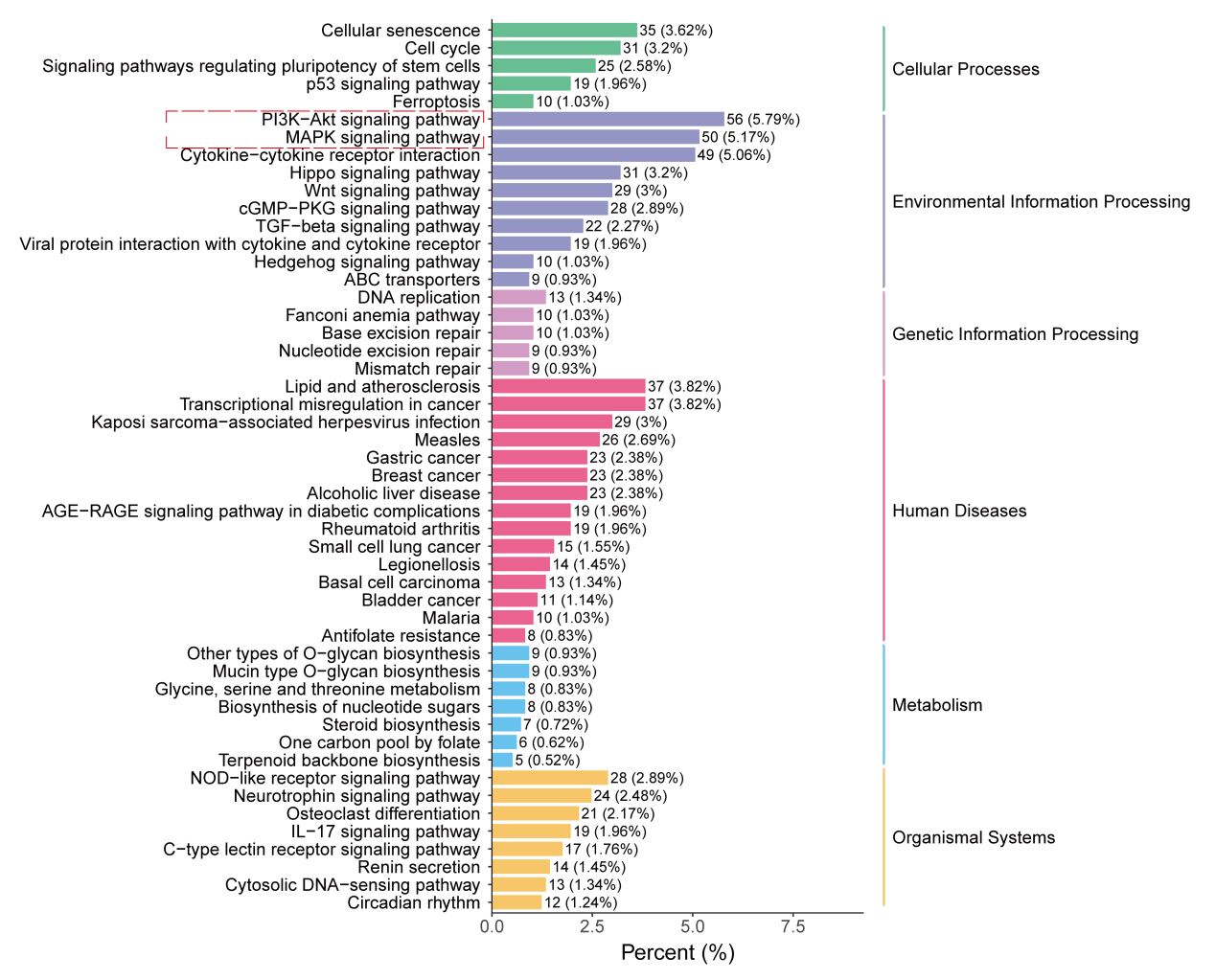


**Fig. S5.** KEGG pathway enrichment analyses on key pathways and biological processes associated with CIr2 sensitivity in MDA-MB-231 cells.


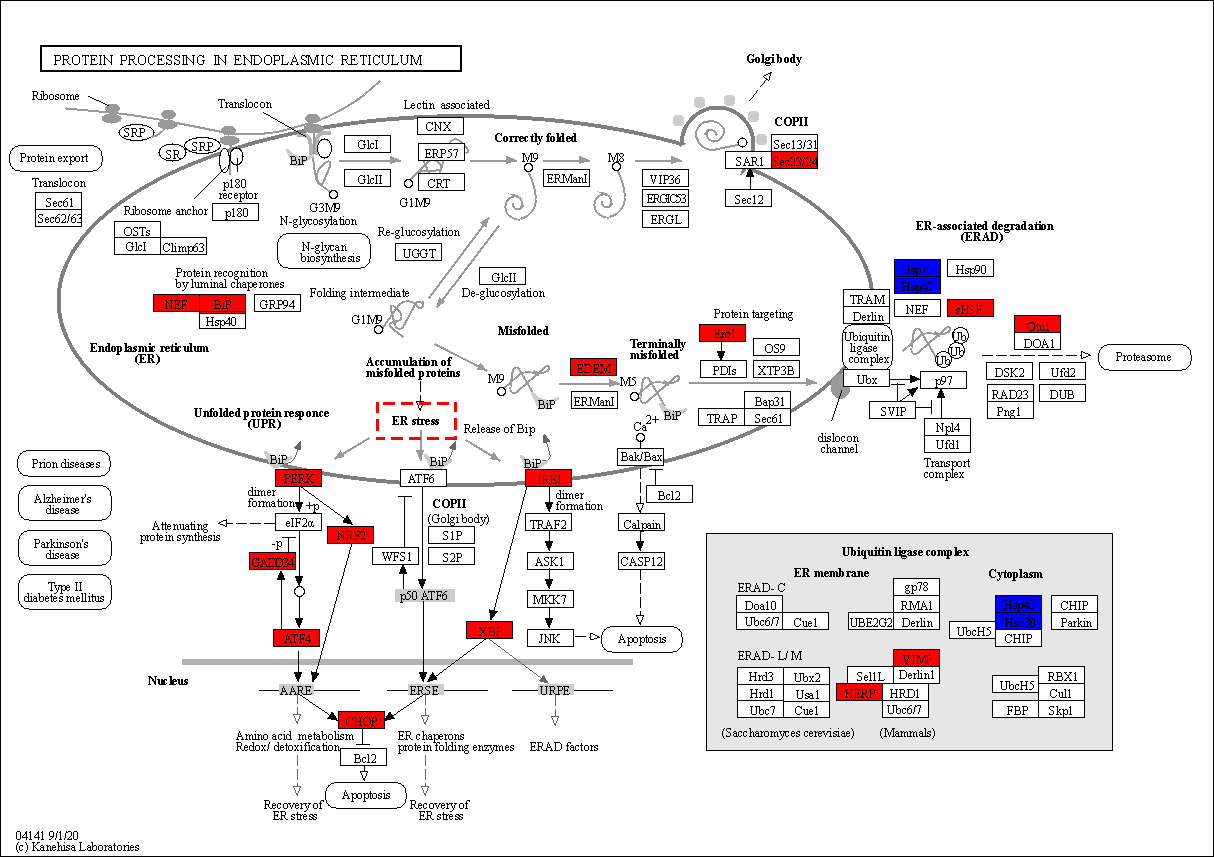


**Fig. S6.** KEGG pathway diagram of protein processing in the endoplasmic reticulum showing differentially expressed genes after CIr2 treatment in MDA-MB-231 cells. Red indicates relative gene up regulation, blue indicates relative gene down regulation.


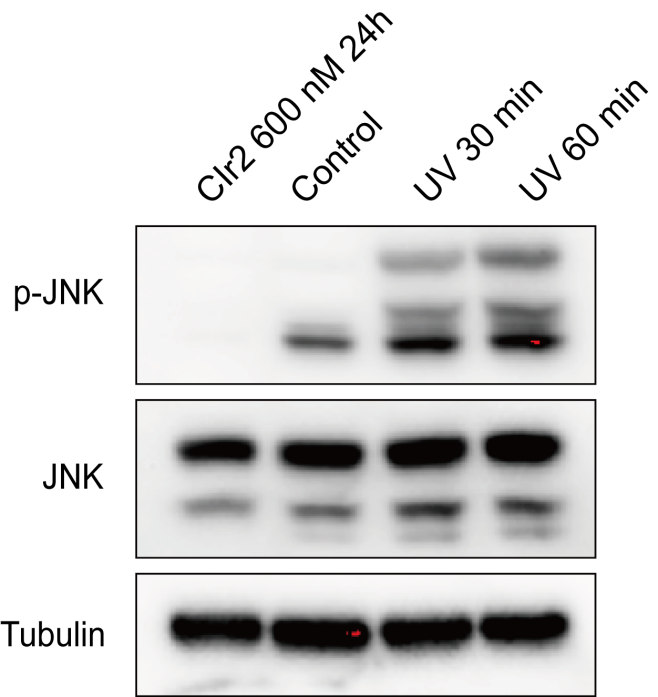


**Fig. S7.** Western blot analysis of p-JNK and JNK in MDA-MB-231 cells treated with CIr2 600 nM for 24 h. Cells treated with UV for 30 and 60 min served as positive controls.


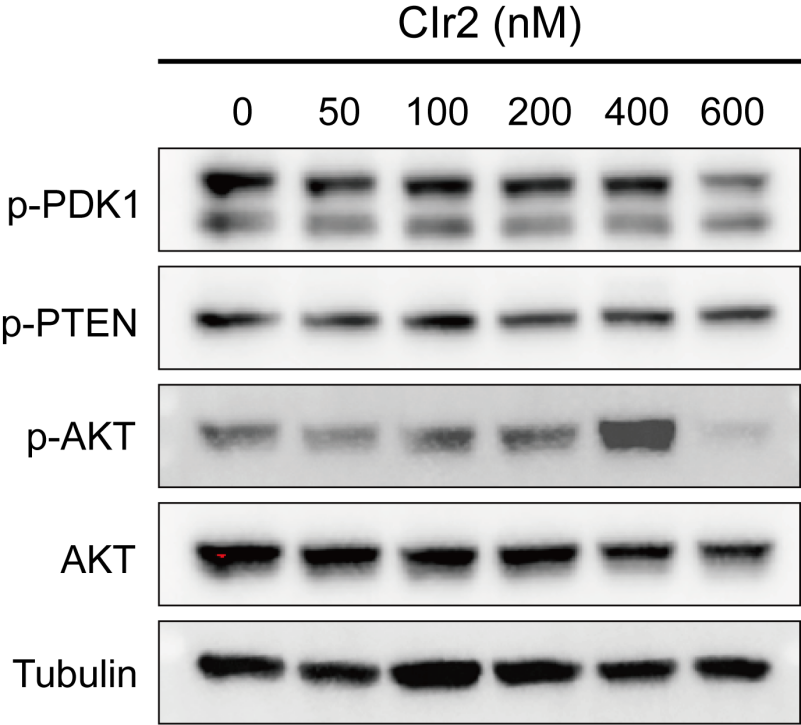


**Fig. S8.** Western blot analysis of cleaved p-PDK1, p-PTEN, p-AKT, AKT after treatment of MDA-MB-231 cells with CIr2 at 50, 100, 200, 400, 600 nM respectively for 24h.


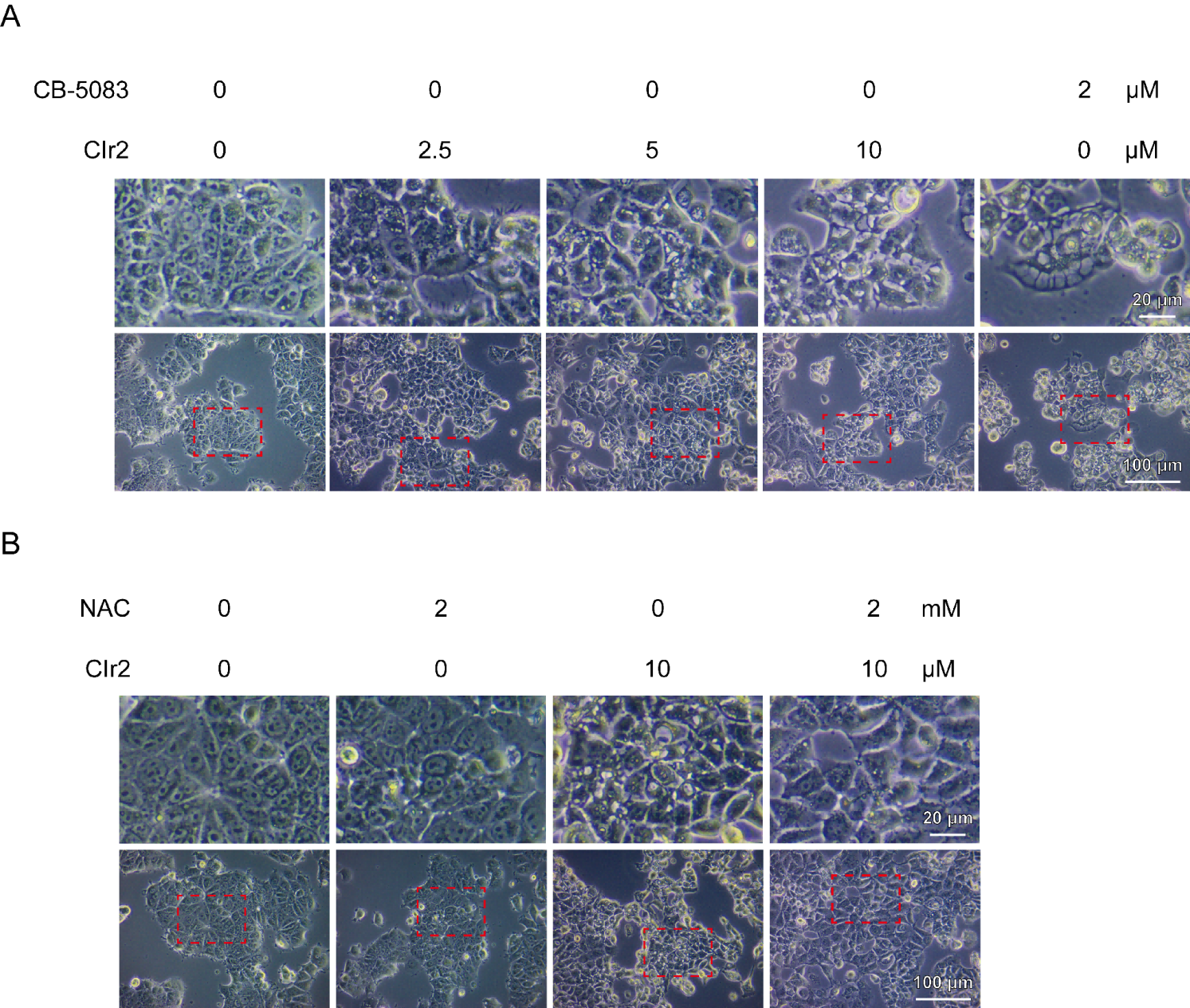


**Fig. S9**. (A) MCF-7 cells were treated with the indicated concentrations of CIr2 or CB-5083 for 24 h, images were obtained using phase-contrast microscope. (B) MCF-7 cells were treated with the indicated concentrations of CIr2 or NAC for 24 h, images were obtained using phase-contrast microscope. Scale bar: 20 μm & 100 μm.


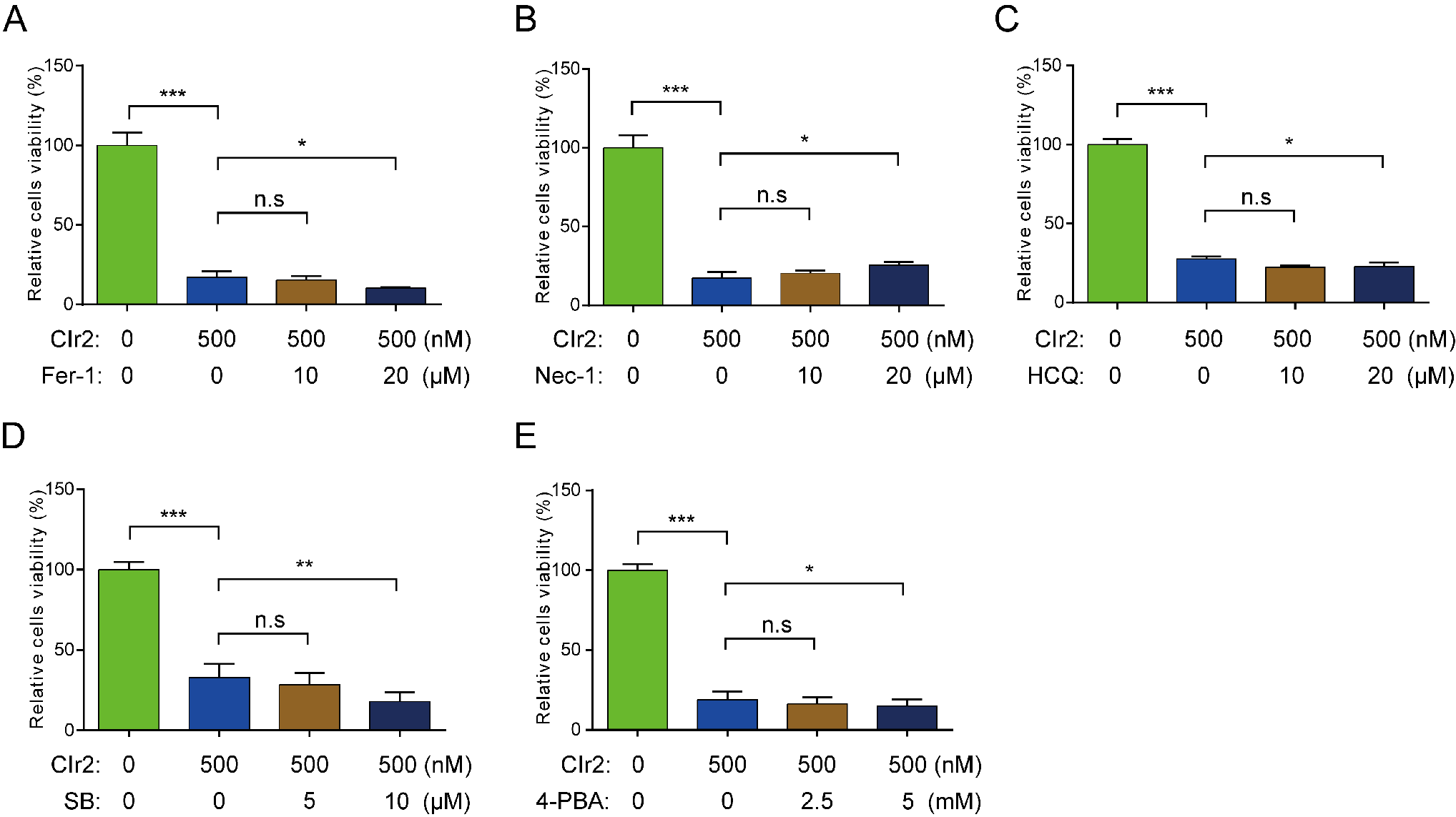


**Fig. S10.** Effect of different inhibitors on the viability of CIr2-treated MDA-MB-231 cells. Cells were treated with CIr2 in the presence of (A) Ferrostatin-1, (B) Necrostatin-1, (C) HCQ, (D) SB202190, or (E) 4-PBA for 24 h. (*p < 0.05, **p < 0.01, ***p < 0.001).
